# Diagnostic Challenges of Syphilis in a Resource-Limited Rural Setting: A Case Report

**DOI:** 10.7759/cureus.109451

**Published:** 2026-05-22

**Authors:** Jose Antonio Medina Sanchez

**Affiliations:** 1 Faculty of Medicine, University of Zulia, Maracaibo, VEN

**Keywords:** public health, rural areas, secondary syphilis, treponema pallidum, vdrl

## Abstract

Syphilis, a sexually transmitted infection caused by *Treponema pallidum*, continues to represent an important public health challenge worldwide, especially in underserved and resource-limited settings. We report a 65-year-old man from a rural area who presented with progressive weight loss, malaise, generalized lymphadenopathy, and a trunk rash, features that can mimic HIV, tuberculosis, viral infections, or lymphoma. A rapid HIV/Syphilis Duo test (SD BIOLINE, Standard Diagnostics Inc., South Korea) was reactive for syphilis and negative for HIV, but no nontreponemal or confirmatory assays were available. According to international guidelines, accurate diagnosis requires both treponemal and nontreponemal testing, yet in rural settings, clinicians often must act on incomplete data. Based on the clinical findings and reactive treponemal test, a presumptive diagnosis of secondary syphilis was made, and benzathine penicillin G 2.4 million units intramuscularly was administered. This case highlights the diagnostic and management challenges faced in resource-limited environments, where overlapping clinical features, incomplete diagnostic algorithms, and restricted access to treatment and follow-up increase the risk of persistent infection and continued transmission.

## Introduction

Syphilis, caused by the spirochete *Treponema pallidum* [1], is a treatable infection that nevertheless remains highly prevalent worldwide. Despite the widespread availability of antibiotics, it continues to represent a major global public health problem [2]. The World Health Organization (WHO) reported nearly eight million new adult cases in 2022. Just two years earlier, it was around 7.1 million [3]. In the Americas, the numbers went up even faster. From 2020 to 2022, adult cases rose by about 30%. In 2022 alone, more than 3.3 million infections were recorded, including more than 68,000 congenital cases [4]. These data demonstrate the persistent and increasing burden of syphilis in the region.

A report estimated a syphilis prevalence of approximately 2.8% among pregnant women in antenatal care in Venezuela, one of the highest rates in Latin America [5]. These findings suggest a persistent risk of ongoing transmission, including congenital syphilis. It also shows how important accurate tests are for vulnerable groups. In Venezuela, years of political and economic crisis have weakened the healthcare system and limited access to diagnostic and treatment services for sexually transmitted infections. Congenital syphilis remains a significant public health concern in Venezuela. A hospital-based study in Barquisimeto reported a 7.3% incidence of congenital syphilis among newborns between 2021 and 2022 [6]. Similarly, a seroprevalence study among blood donors in Maracaibo between 2012 and 2014 found that 2.95% had antibodies to *Treponema pallidum*, indicating prior or current exposure to syphilis [7]. These data highlight the persistent burden of syphilis in the country, despite its preventable nature.

Syphilis demonstrates a broad clinical spectrum that varies according to disease stage, ranging from localized painless ulcers and regional lymphadenopathy in primary infection to systemic manifestations such as diffuse rash, constitutional symptoms, mucocutaneous lesions, and generalized lymphadenopathy in secondary disease. Latent infection may remain clinically silent for years, while untreated late-stage disease can result in cardiovascular, neurologic, and gummatous complications. Because these manifestations overlap with multiple infectious, inflammatory, and malignant conditions, syphilis is frequently referred to as "the great imitator" [2,8].

Diagnosis needs both a nontreponemal test (venereal disease research laboratory (VDRL), rapid plasma reagin (RPR)) and a treponemal test. Nontreponemal titers are required for staging and follow-up. Treponemal rapid diagnostic tests, including dual HIV/syphilis assays, are useful for screening in rural areas. But these tests cannot show whether the infection is active or old [1].

This diagnostic challenge is particularly evident in rural regions of Venezuela such as Sinamaica, La Guajira, where geographic isolation, limited laboratory infrastructure, shortages of medical supplies, and barriers to transportation restrict access to confirmatory testing and specialist referral centers. In these settings, clinicians frequently rely on rapid treponemal tests and clinical judgment alone, increasing diagnostic uncertainty and complicating staging, treatment monitoring, and follow-up.

We present the case of an older sexually active man from Sinamaica who had generalized lymphadenopathy and a reactive treponemal rapid test. The case underlines the uncertainty doctors face when only rapid tests are available. It also points to the public health risk of delayed or missed treatment in underserved rural areas.

## Case presentation

A 65-year-old sexually active man from Sinamaica, a rural town in Venezuela, presented to the local clinic with several weeks of progressive weight loss, persistent fatigue, and malaise. Approximately two weeks after symptom onset, he developed multiple spots on his trunk that gradually became more numerous, spreading across the chest and back. His past medical history was unremarkable, with no known chronic diseases, previous hospitalizations, or history of sexually transmitted infections. He had not traveled outside the region recently. The patient reported multiple female partners with inconsistent condom use but denied fever, cough, night sweats, alcohol consumption, and illicit drug use.

**Figure 1:**
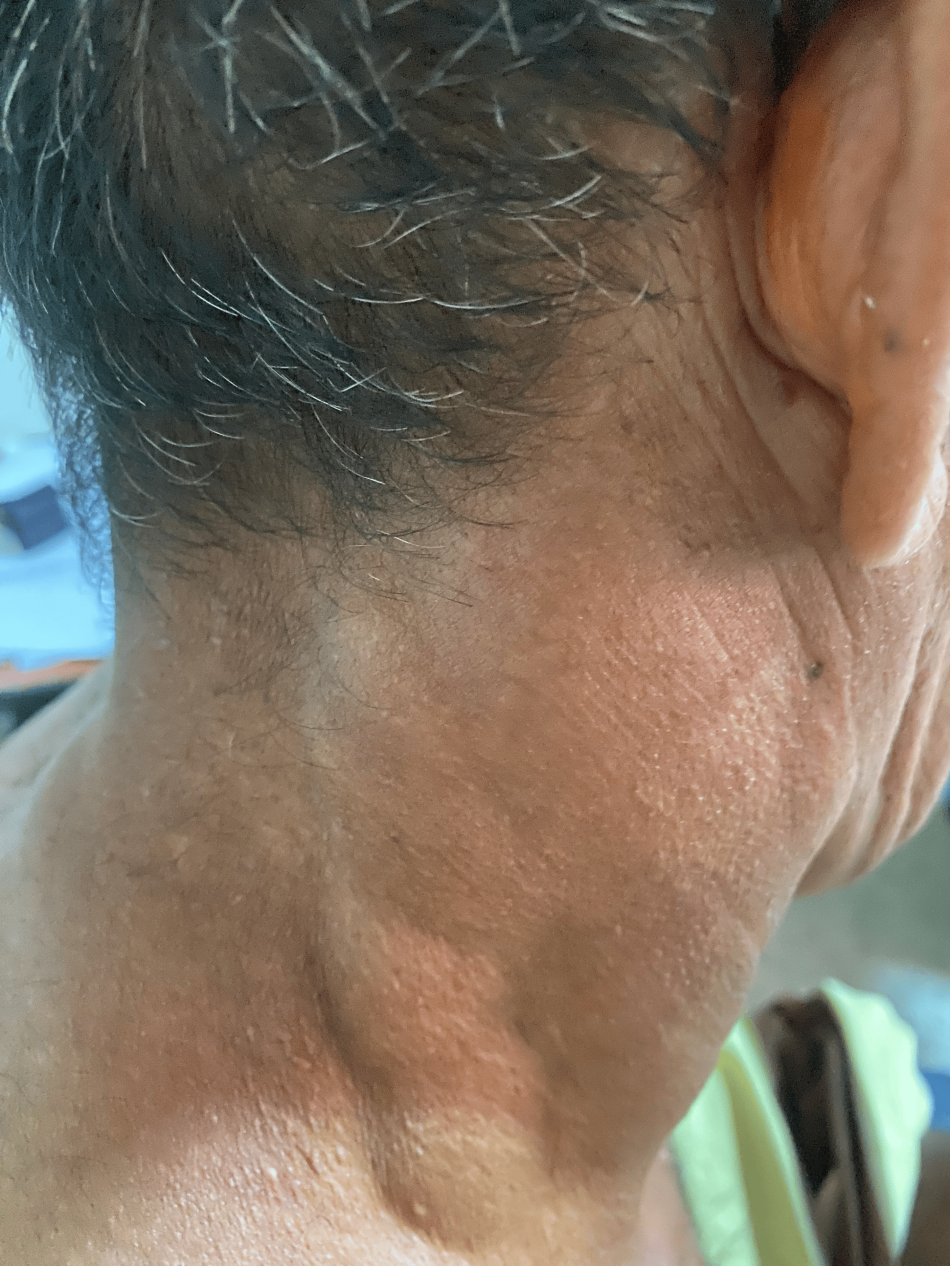
Cervical lymphadenopathy (a firm, non-tender, mobile node located on the right side)

**Figure 2:**
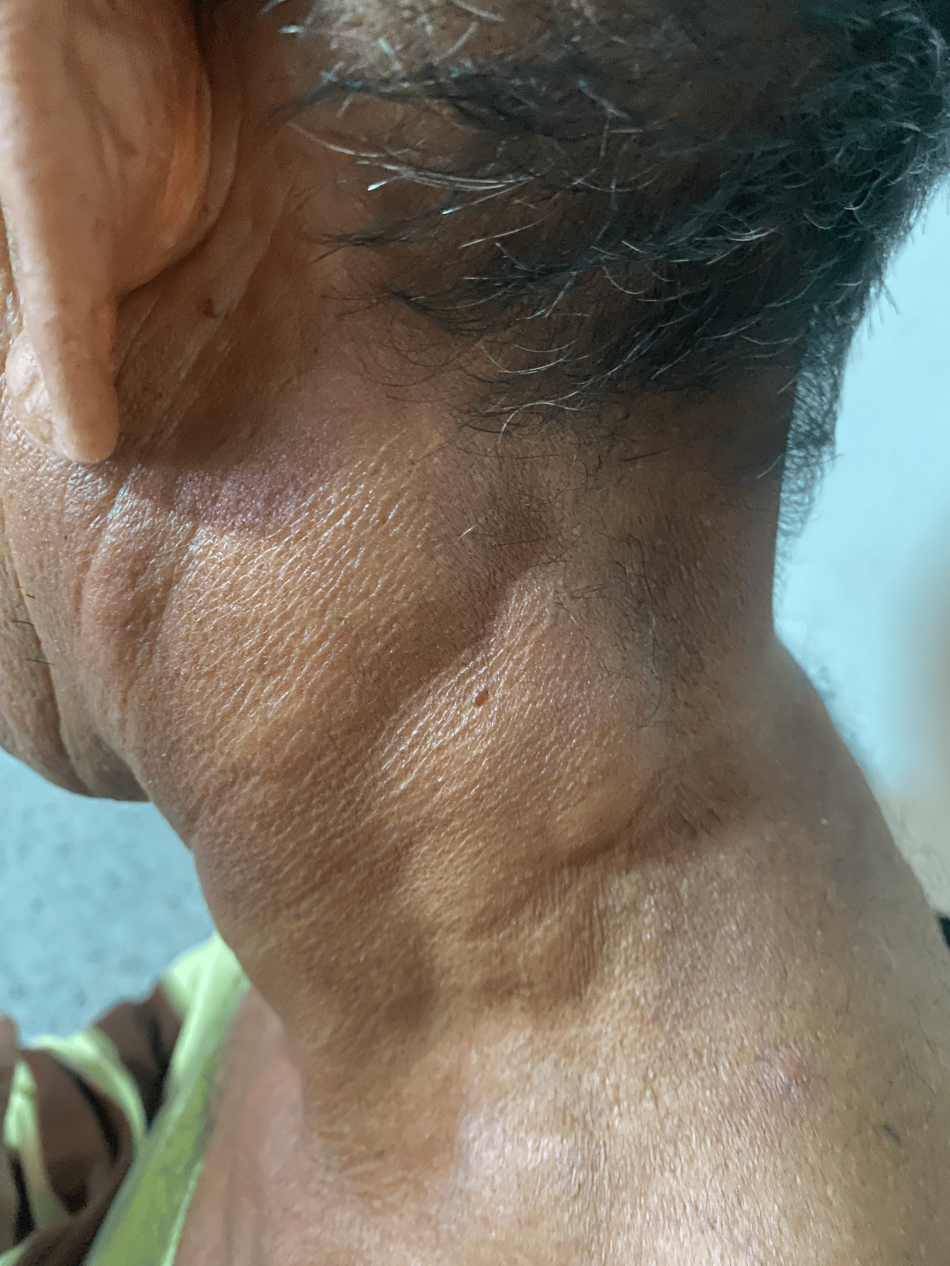
Cervical lymphadenopathy (a firm, non-tender, mobile node located on the left side)

**Figure 3:**
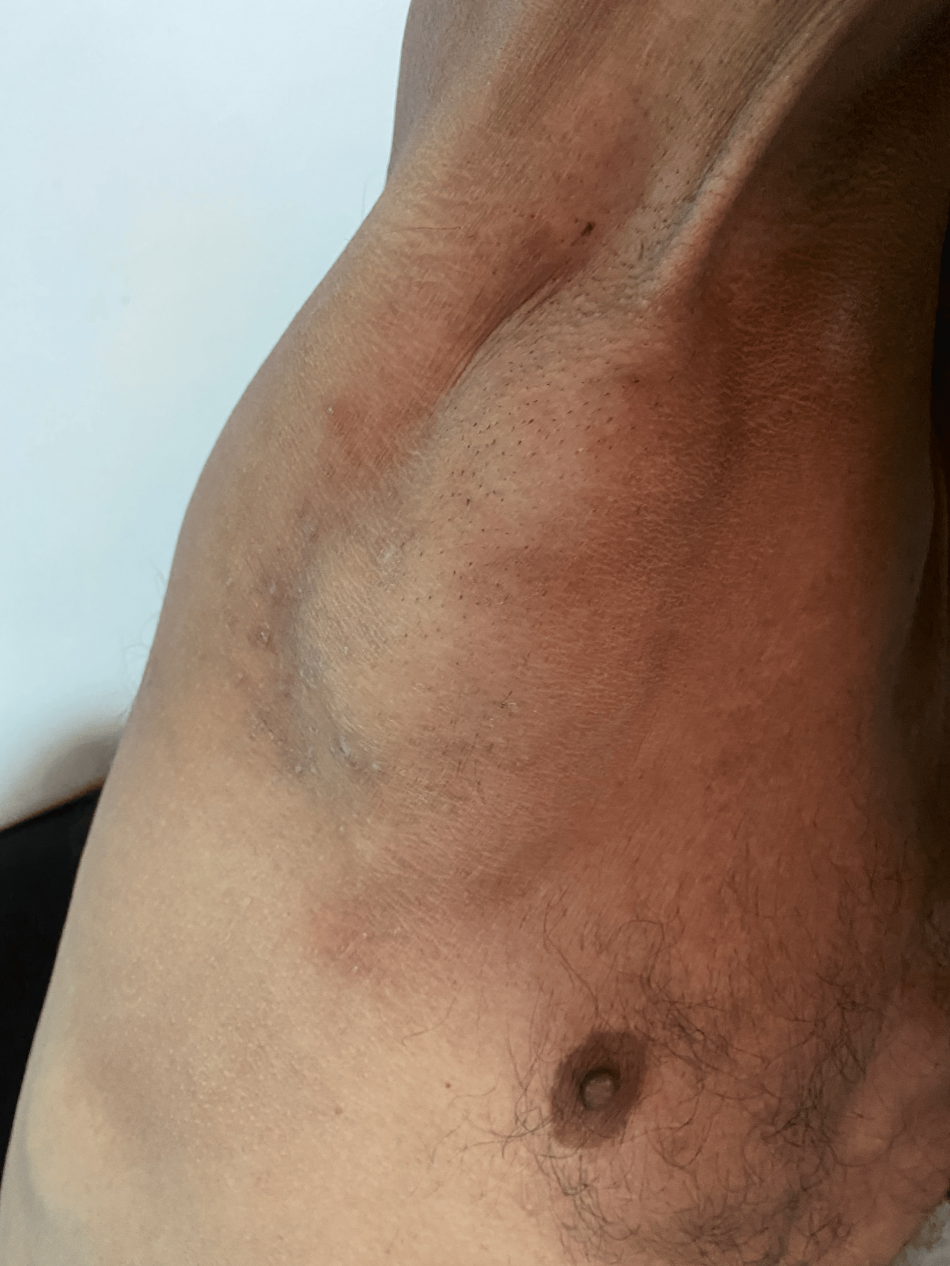
Axillary lymphadenopathy (an enlarged, non-tender node located on the right side)

**Figure 4:**
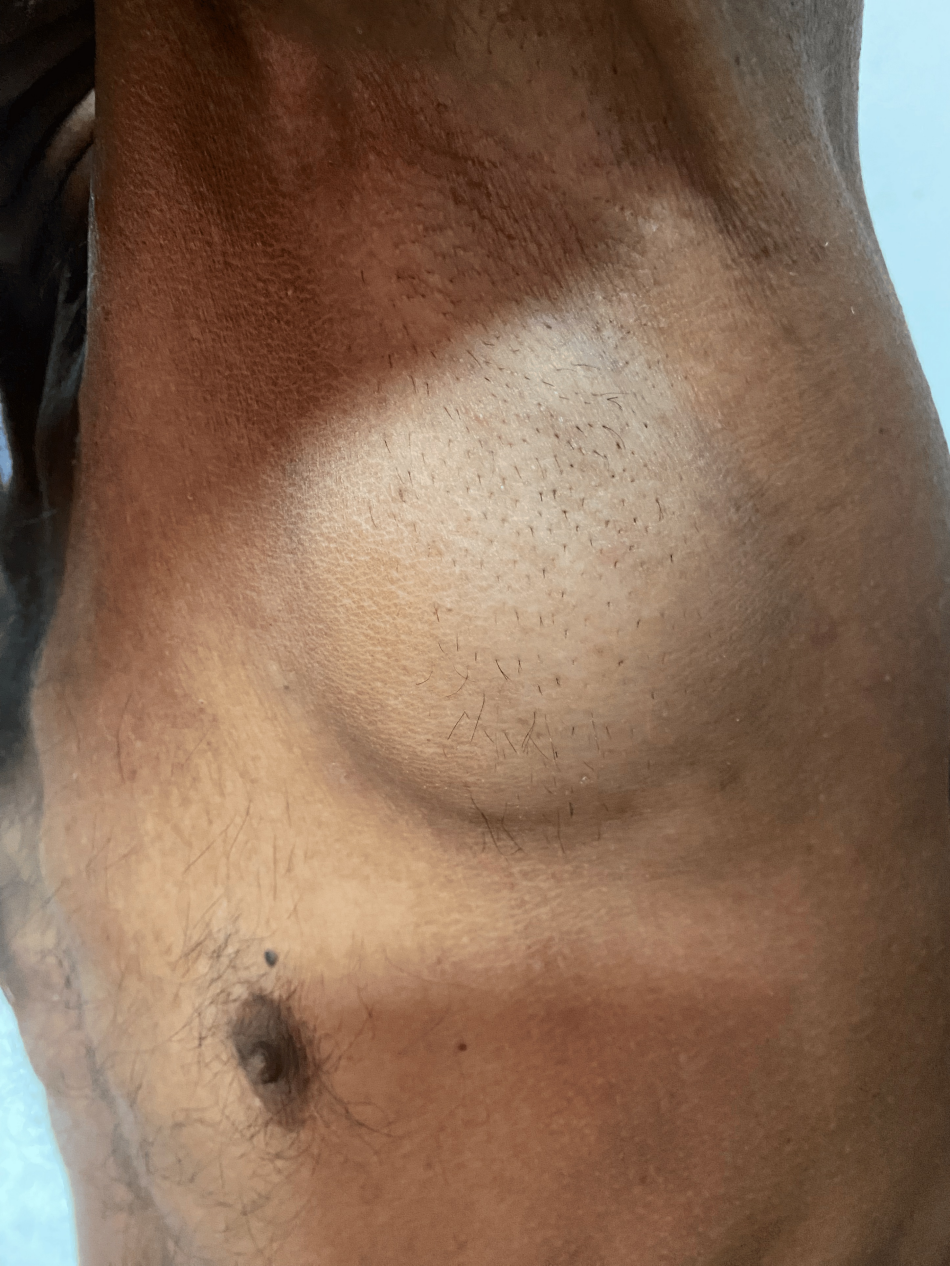
Axillary lymphadenopathy (an enlarged, non-tender node located on the left side)

**Figure 5:**
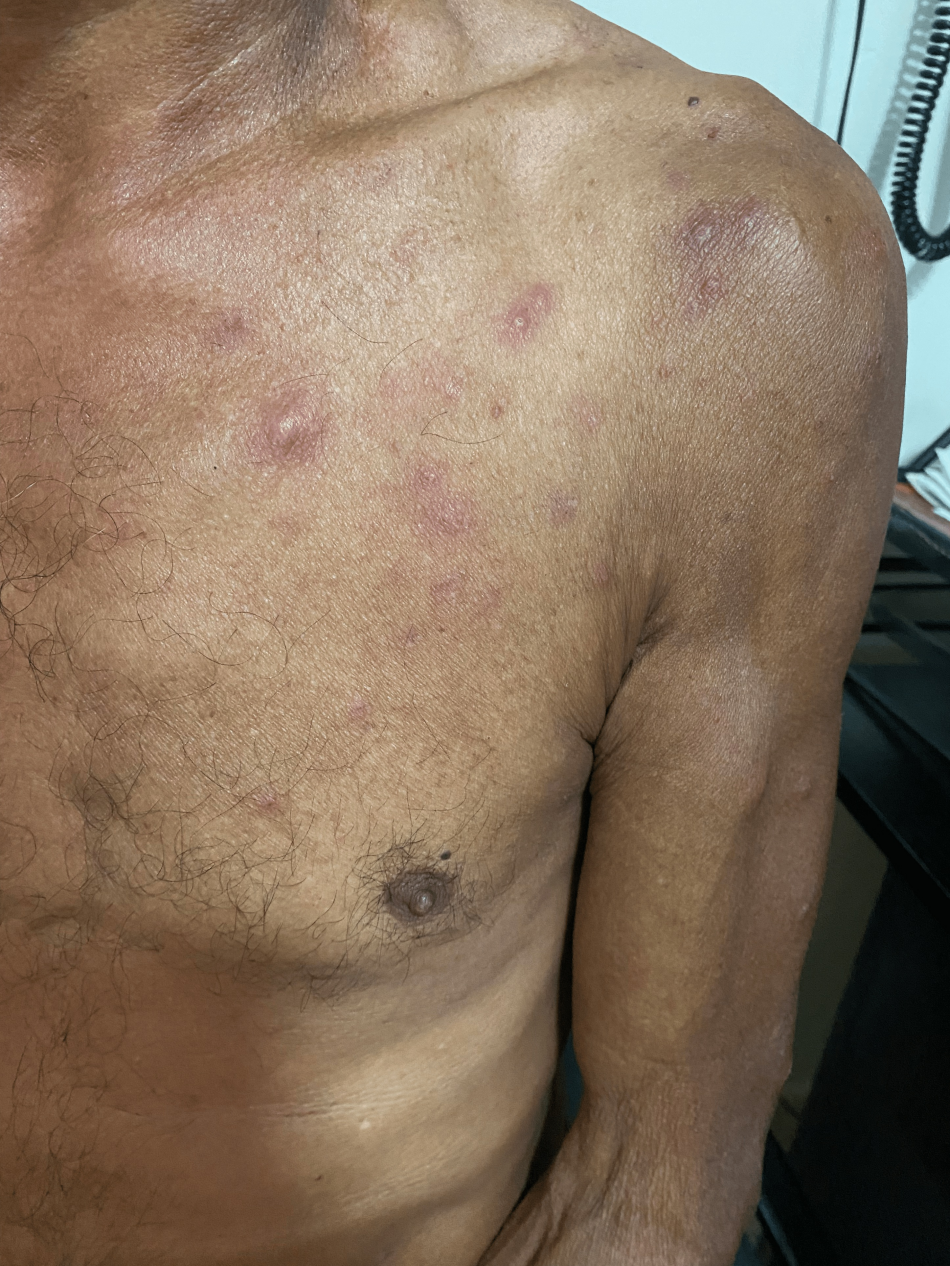
Non-pruritic maculopapular lesions on the trunk

On physical examination, he appeared comfortable and afebrile. Vital signs were stable: blood pressure 122/78 mmHg, heart rate 80 beats per minute, respiratory rate 18 per minute, and oxygen saturation 98% on room air. Lymphadenopathy was generalized. The nodes were firm, mobile, and not tender to palpation. They were palpable bilaterally in the cervical and axillary regions. The largest nodes measured roughly 4-5 cm. The skin exam showed multiple erythematous to brown maculopapular lesions. They measured between 0.5 and 1 cm. They were scattered across the trunk. The lesions were not itchy. There was no ulceration, no scaling, or no mucosal lesions (Figures 1-5).

A point-of-care HIV/Syphilis Duo rapid test (SD BIOLINE, Standard Diagnostics Inc., South Korea) was performed. The treponemal line was positive. The HIV line was negative. No other testing could be performed at the time. In this rural setting, no RPR or VDRL was available. Confirmatory HIV testing and imaging studies were not available either. The clinic serves a wide, scattered population with poor transportation. Patients often cannot reach referral hospitals. Financial barriers are frequent. These factors limit diagnostic workup and also reduce the chances of structured follow-up.

The differential diagnosis included secondary syphilis, HIV, tuberculosis, lymphoma, and viral illnesses. HIV was unlikely given a non-reactive rapid test. Tuberculosis was excluded by the absence of cough, fever, or night sweats. Lymphoma was less likely without B symptoms such as fever or drenching sweats. Viral infections (Epstein-Barr virus/cytomegalovirus (EBV/CMV)) were improbable as there was no pharyngitis or hepatosplenomegaly. The presentation of non-tender generalized lymphadenopathy with a maculopapular rash was most consistent with secondary syphilis. A presumptive diagnosis of secondary syphilis was made on the basis of clinical features and the reactive treponemal test. In accordance with international guidelines, the patient was administered benzathine penicillin G 2.4 million units intramuscularly in a single dose. Referral for confirmatory testing and follow-up was discussed; however, the likelihood of structured follow-up was considered low due to socioeconomic barriers. At the time of last contact, the patient had not returned.

## Discussion

Despite the availability of effective treatment, syphilis remains difficult to control because a substantial proportion of infections are asymptomatic or overlooked, allowing continued transmission within vulnerable populations. Secondary syphilis frequently manifests with diffuse non-pruritic rash, generalized lymphadenopathy, and systemic symptoms including weight loss and malaise. These features match the presentation of our patient who was sexually active. WHO emphasizes that rapid tests provide results within minutes and can enable treatment initiation at the same visit, although they do not replace conventional tests for staging and follow-up [9].

In this case, the absence of nontreponemal testing limited the ability to determine disease activity, establish clinical staging, and monitor treatment response. This reflects the broader diagnostic challenges faced in rural Venezuela, where clinicians frequently depend on treponemal rapid assays because of restricted laboratory infrastructure and limited access to confirmatory testing. Nontreponemal tests are quantitative, correlate with disease activity, and are necessary for staging and monitoring treatment response. In contrast, treponemal tests are highly specific but remain positive for life, even after successful therapy, and therefore cannot distinguish between active and past infection [1].

Although both traditional and reverse diagnostic algorithms are widely used for syphilis screening, their effectiveness in rural settings is often limited by restricted access to complementary testing. In this case, reliance on a treponemal rapid assay without nontreponemal confirmation limited accurate staging and treatment monitoring. Additionally, treponemal rapid tests may remain positive after prior treated infection and may produce false-negative results in early disease, highlighting the risks of overreliance on point-of-care testing alone in resource-limited environments. Both approaches require access to both test types to provide accurate diagnosis and treatment monitoring [1]. However, in rural environments such as Sinamaica, treponemal rapid tests are typically the only tests available, meaning clinicians must act on incomplete data.

In secondary syphilis, generalized lymphadenopathy and trunk rash are common, but they overlap with HIV, EBV/CMV, tuberculosis, and lymphoma; several case reports show secondary syphilis closely mimicking lymphoma on imaging and clinical grounds, delaying diagnosis when serology is incomplete [10].

If untreated, syphilis progresses through latent stages and may eventually cause cardiovascular, neurologic, and gummatous complications, contributing to long-term morbidity and mortality. In addition, untreated sexually active adults remain reservoirs for ongoing transmission, including congenital syphilis in pregnant women [9]. The WHO estimates that in the Americas, congenital syphilis continues to increase in parallel with rising adult incidence. This represents a major public health risk in underserved rural communities [4]. Loss to follow-up is another critical issue in these environments. Patients frequently cannot return due to transportation costs, geographic isolation, or socioeconomic barriers. As a result, untreated individuals remain in the community, fueling further transmission and undermining control efforts.

International guidelines provide specific direction for such cases. The Centers for Disease Control and Prevention (CDC) emphasizes that presumptive treatment is warranted when a patient presents with compatible clinical features and a reactive treponemal test in settings where nontreponemal assays are unavailable [1]. Benzathine penicillin G remains the recommended treatment [9]. Without treatment and structured follow-up, both the individual and the wider community remain at risk.

Diagnosing syphilis in low-resource rural settings is challenging because the full laboratory algorithm recommended by international guidelines from the CDC and WHO is often unavailable. In this case, clinicians had to rely on a single treponemal rapid test and compatible clinical features, without the possibility of confirmatory staging or structured follow-up. This reflects the broader challenges of managing syphilis in resource-constrained environments, where incomplete diagnostic tools, limited access to treatment, and loss to follow-up increase the risk of untreated infection and continued community transmission. Addressing these gaps requires strengthening supply chains for diagnostics and essential medicines, as well as community-based strategies to improve follow-up and partner management.

## Conclusions

This case underscores the diagnostic and management difficulties of syphilis in rural, resource-limited settings. When only treponemal rapid tests are available, clinicians must often rely on incomplete data, which prevents staging, monitoring, and confirmation of infection. In our patient, generalized lymphadenopathy, weight loss, and a reactive treponemal test were highly suggestive of secondary syphilis, but the absence of nontreponemal assays and confirmatory testing limited diagnostic certainty.

Syphilis remains a significant public health concern in Venezuela, with high prevalence in antenatal populations and rising congenital cases. Untreated infection not only endangers individual health through late complications but also sustains community transmission, particularly where follow-up is poor. International guidelines recommend presumptive treatment under these circumstances, when clinical suspicion and a reactive treponemal test cannot be further confirmed with standard serology, yet barriers to drug access and fragile health infrastructure continue to hinder appropriate care. Strengthening diagnostic capacity, ensuring reliable access to benzathine penicillin G, and implementing community-level strategies for follow-up are essential steps to reduce the burden of syphilis in underserved populations.
